# Strategies for investigating the maternal-fetal interface in the first trimester of pregnancy: What can we learn about pathology?

**DOI:** 10.1016/j.placenta.2017.05.003

**Published:** 2017-12

**Authors:** Judith E. Cartwright, Guy StJ. Whitley

**Affiliations:** Molecular and Clinical Sciences Research Institute, St. George's, University of London, Cranmer Terrace, London, SW17 0RE, UK

**Keywords:** First trimester, Decidua, Placenta, Uterine artery doppler screening, Pre-eclampsia

## Abstract

The pathologies of the pregnancy complications pre-eclampsia (PE) and fetal growth restriction (FGR) are established in the first trimester of human pregnancy. In a normal pregnancy, decidual spiral arteries are transformed into wide diameter, non-vasoactive vessels capable of meeting the increased demands of the developing fetus for nutrients and oxygen. Disruption of this transformation is associated with PE and FGR. Very little is known of how these first trimester changes are regulated normally and even less is known about how they are compromised in complicated pregnancies. Interactions between maternal and placental cells are essential for pregnancy to progress and this review will summarise the challenges in investigating this area. We will discuss how first trimester studies of pregnancies with an increased risk of developing PE/FGR have started to provide valuable information about pregnancy at this most dynamic and crucial time. We will discuss where there is scope to progress these studies further by refining the ability to identify compromised pregnancies at an early stage, by integrating information from many cell types from the same pregnancy, and by improving our methods for modelling the maternal-fetal interface *in vitro*.

## Highlights

• Pathology of PE/FGR begins in the first trimester.
• Investigating pregnancies with increased risk of PE/FGR is giving valuable information.
• This will improve further with advances in identifying compromised pregnancies.

## 1 Introduction

The events that occur at the maternal-fetal interface define the success of a pregnancy yet are the most challenging to study. Trophoblast invasion and remodelling of the uterine vasculature occur in the first trimester of pregnancy and are tightly regulated in both a temporal and spatial manner. Information on the maternal-fetal interface in the first trimester can be gained from immunohistochemical studies however this does not give an indication of which dynamic cellular and molecular interactions are of functional importance in regulating these crucial events. In this review we will discuss some of the strategies taken to investigate the field, how they have informed what we know about the cell biology of normal pregnancies and those with placental complications and where the scope for future development lies.

## 2 Regulation of trophoblast invasion and spiral artery remodelling

Trophoblast invasion occurs in a tightly regulated manner, influenced by signals from the surrounding decidual environment. During the decidualisation process maternal immune cells are recruited to the uterus in significant numbers, with approximately 70% being decidual natural killer (dNK) cells, 20% macrophages and the remaining 10% T cells. During placentation, extravillous trophoblast (EVT) cells invade into the decidua and interact with this enriched environment of specialised immune cells. Cross-talk between the immune cells, stromal cells and the fetal EVT will contribute to regulating EVT invasion and the accompanying changes to the spiral artery structures required for successful pregnancy.

Spiral artery remodelling involves the loss of vascular cells and matrix proteins and occurs in an orchestrated fashion. The initial steps involve activation and vacuolisation of endothelial cells (EC), hypertrophy and disorganisation of vascular smooth muscle cells (VSMC) and fibrinoid deposition [1] while complete remodelling requires EVTs [2]. Dramatic changes to the architecture of the spiral arteries occur as they remodel in the first trimester with cellular events identified including: apoptosis of vascular cells, cellular migration, de-differentiation and altered secretion of matrix degrading enzymes. Following successful remodelling the resulting high-flow, low-resistance vessel is then capable of supplying sufficient blood to the intervillous space to meet the increasing demands of the developing fetus. Failure of spiral artery remodelling is an early characteristic of common pregnancy complications such as fetal growth restriction (FGR) and pre-eclampsia (PE) [3].

EVT invasion can be either interstitial or endovascular with endovascular EVTs interacting directly with the EC while interstitial EVT interact first with the VSMCs. Invasion by either route will influence vascular structure via disruption of the heterotypic interactions between EC and VSMC known to be important for maintaining a healthy differentiated vessel [4]. For example, damage to the EC layer has been shown to results in VSMC de-differentiation in other vessels [5]. It is noteworthy that even in pregnancies with poor remodelling, trophoblasts plugs are present and both endovascular and interstitial EVTs are seen. This would suggest that failure to remodel cannot be explained by poor invasion alone but also requires a failure of EVTs to interact appropriately with the vascular cells.

There is increasing evidence that maternal immune cells in addition to the fetal EVT play important roles in the remodelling process. In humans all of the immune cell types are located around actively remodelling vessels [6]. It is suggested that the immune cells are involved in the changes that occur before the arrival of trophoblast as well as regulating trophoblast-dependent remodelling [6], [7], [8], [9], [10], [11], [12]. For example leukocyte disruption of both EC and VSMC was apparent before EVT were present [6] and processes involving matrix degradation, vascular cell apoptosis and de-differentiation have been implicated [11].

The ability to investigate during the timeframe in which these changes are actively occurring is hindered by the difficulties inherent in studying human pregnancy. A wide-ranging evaluation of the cellular and molecular cross-talk at the maternal-fetal interface, at what is the most critical time in the first trimester, therefore remains to be performed. Being able to develop strategies to try and address this would advance our understanding of both normal human pregnancy as well as pregnancies complicated by PE and FGR.

## 3 When can we study the maternal-fetal interface?

**Fig. 1:**
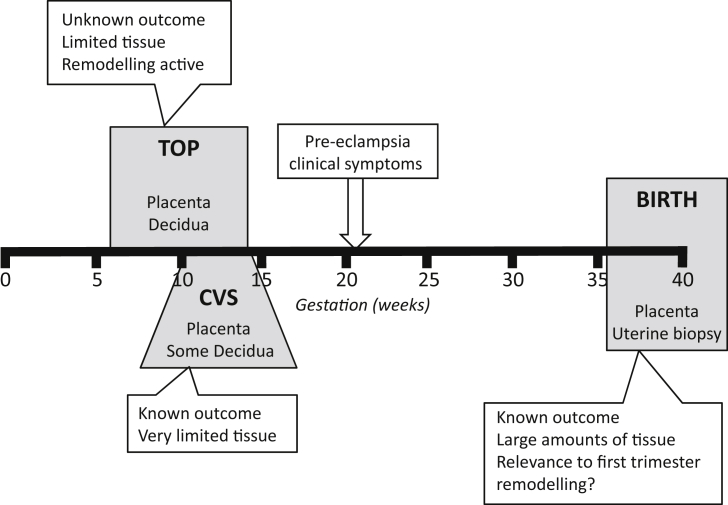
**Tissue accessibility at different stages of pregnancy.** The tissue available at termination of pregnancy (TOP), chorionic villous sampling (CVS) or at parturition (birth) are shown (grey boxes). The limitations and advantages of what can be studied from tissue obtained at these times is summarised (white boxes). Pre-eclampsia is shown as being detected clinically after approximately 20 weeks gestation.

Investigating vascular changes at a molecular or cellular level in early human pregnancy is restricted by ethical issues and while animals model some aspects of human pregnancy, they lack deep trophoblast invasion and comparable spiral artery transformation [13]. Some of the challenges in obtaining tissue are highlighted in Fig. 1 on a timeline of gestation. All of these types of tissue can be informative at different stages of pregnancy and have advantages and disadvantages in what can be extrapolated from them regarding events occurring in both normal and pathologic pregnancy.

Access to human tissue is limited in ongoing pregnancies to that obtained during chorionic villous sampling (CVS) for genetic screening in the first to early second trimester, or that obtained following parturition. Some highly informative gene array studies have been carried out on tissue obtained from CVS suggesting that the decidual environment may be key in the establishment of placental pathology [14], [15]. A considerable advantage of these studies is that the pregnancy can be followed to determine whether the woman develops any pregnancy complications or has a normal pregnancy outcome [16]. The amount of tissue obtained is however very small making it less amenable for cellular studies. Going forward this may not continue to be a feasible approach as the use of chorionic villous sampling as a diagnostic technique is diminishing due to the increasing use of non-invasive prenatal testing [17].

Placental tissue obtained at term comes from a pregnancy where it is known whether the woman developed PE or another placental complication and has the advantage that tissue is plentiful as the whole placenta can be studied. However this represents a time many months after active remodelling has occurred and any differences in the placentas of PE/FGR pregnancies may represent a consequence of the disorder rather than inform causative pathology. To investigate the decidua and myometrium, uterine biopsies can be taken at the time of delivery and histochemical studies carried out or cells and vessels isolated. This approach has proven useful in developing models of spiral artery remodelling where non-placental bed vessels are obtained and used in co-culture with EVT [18], [19]. These vessels will not have been in contact with EVT during pregnancy so can help model some aspects of the remodelling process however they will have been changed by the non-EVT dependent remodelling [1] so are not completely representative of the pre- and early pregnancy vessels.

Tissue samples can also be obtained following termination of pregnancy in the first trimester. The approach taken by our group and others has been to focus on events occurring in the first trimester as this is the most informative time to study EVT invasion and vessel remodelling. Most studies using first trimester tissue have been done without any additional information to inform whether that pregnancy was developing normally. Herein lies the challenge as the clinical symptoms of conditions such as PE are not apparent until after 20 weeks gestation. How can we look at first trimester tissue and know whether it is from a normal or pathologic pregnancy? We have started to address this by using uterine artery resistance index (RI) measurements obtained by Doppler Ultrasound screening to identify women who would have had an increased risk of pregnancy complications had the pregnancy progressed, allowing us to divide the first trimester placental and decidual tissue into two study groups; that from pregnancies with a higher risk and a very low risk of developing PE/FGR. A disadvantage of this approach lies in the fact that many of the pregnancies designated higher risk in the first trimester would have ended up progressing normally despite the probable initial impairment. This will be addressed in the future studies section below. Even given this limitation we have reported fundamental differences in the behaviour of cells isolated from the fetal-maternal interface of high resistance index (RI) compared to normal RI pregnancies which will be described in the following section.

## 4 First trimester studies of pregnancies with an increased risk of developing PE/FGR

Studies of approximately 10,000 ongoing pregnancies showed that cases with a resistance index obtained from uterine artery Doppler scanning of >95th percentile (high RI) had a 5-fold increased risk of that pregnancy developing placental complications compared to those with a normal RI (<95th percentile) [20]. We have used this as a proxy measure of poor remodelling in the first trimester to separate tissue from first trimester terminations of pregnancy into normal RI (normal remodelling) and high RI groups (poor remodelling). With advances in the development of techniques to isolate cells from tissue with high purity and viability we have been able to begin a more comprehensive profiling of these pregnancies.

We have shown that primary trophoblast cells isolated from the hRI pregnancies are more sensitive to apoptotic cell death than those from nRI pregnancies, and this may be a contributing factor to impaired invasion [21]. Additionally when primary EVT were grown out from chorionic villous explant cultures there were less extensive outgrowths from the hRI group compared to the nRI group (unpublished data). Additional evidence for impaired invasion/remodelling comes from immunohistochemical studies showing that there are fewer trophoblast plugs present in the spiral arteries of the hRI group than nRI group [22]. While we have yet to determine factors responsible for this, gene expression profiling of placental tissue from these groups has shown differential expression of genes related to immune and inflammatory responses [20].

Differences in how the maternal immune cells behave between these patient groups is also starting to become clearer with dNK cells from pregnancies with impaired remodelling showing abnormalities in secreted proteins and functional interactions with other decidual cells [7], [8], [23], [24], [25]. For example, dNK cells from nRI pregnancies promote more trophoblast chemotaxis, motility and invasion than those from hRI pregnancies [24], and dNK from hRI pregnancies are less able to activate some of the pro-invasive signalling pathways in trophoblast such as ERK1/2 and Akt [24]. We have demonstrated that the expression of receptors involved in regulating dNK cell effector function differs in the patient groups with KIR2DL/S1,3,5 and LILRB1 (ILT-2) positive dNK cells decreased in pregnancies with a hRI [25]. While the functional outcome of this remains to be determined, at least in the case of LILRB1, this leads to a difference in dNK cell expression of factors that can regulate trophoblast migration/invasion [24].

There is evidence for the involvement of dNK cells directly in spiral artery remodelling [6], [10], [11], [26]. We have shown that dNK cells can induce vascular cell apoptosis and destabilise vessel structures, events of importance in spiral artery changes and that dNK cells from the hRI pregnancies were less able to do this [7], [8].

It is clear that studying the uterine artery Doppler screened patient groups, combined with improved techniques for isolating pure cell types and modelling events *in vitro*, has increased our knowledge of how placentation in the first trimester develops normally and pathologically. There is however scope for much development of these areas in the future, which will be addressed in the following section.

## 5 Future directions

**Fig. 2:**
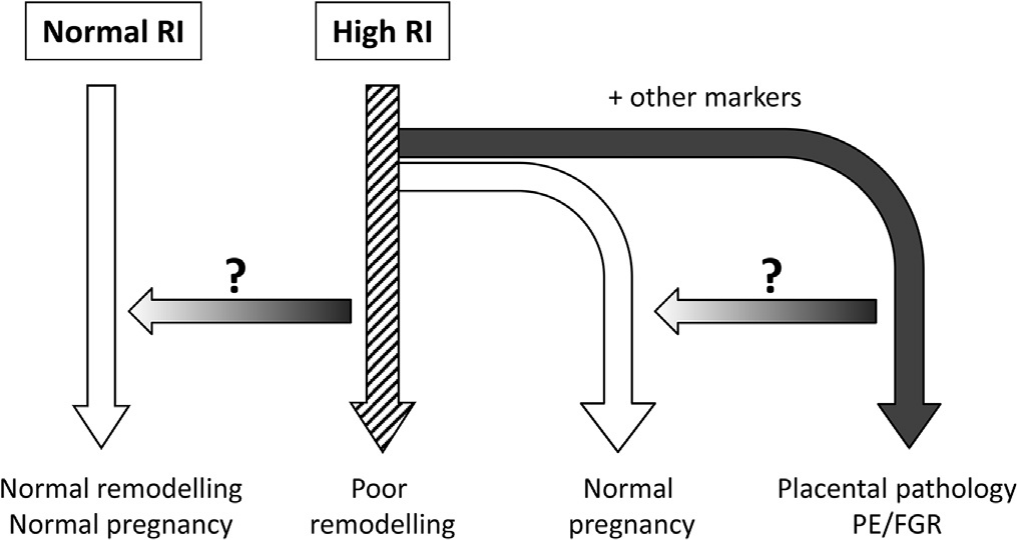
**Scope for further refinement of the uterine artery Doppler screened first trimester patient groups.** Women with a first trimester uterine artery Doppler resistance index (RI) > 95th percentile (high RI) had a 5-fold increased risk of that pregnancy developing placental complications of pregnancy compared to those with a normal RI (<95th percentile). We have used this as a proxy measure of poor remodelling in the first trimester to separate tissue from first trimester terminations of pregnancy into normal RI (normal remodelling) and high RI groups (poor remodelling). However even in the high RI group the majority of these women would not develop pregnancy complications had the pregnancy progressed. With the addition of other markers to further define the high RI group we will be able to investigate how an initial impairment in remodelling can be compensated for. In addition we can start to define the relative contribution of placental/decidual differences and how much of the endpoint is determined by the maternal response to a stressed placenta. The arrows with queries represent areas for possible therapeutic strategies to reverse the pathology once more is understood regarding the cellular and molecular events.

Improvements in early diagnostic tools are proving valuable clinically, for example reliable serum or metabolic biomarkers that more accurately predict development of PE before the clinical symptoms become apparent [27]. Adding supplementary criteria to the uterine artery Doppler screening to classify the first trimester tissue we have described above, would much improve the ability to predict PE risk. This raises the intriguing possibility of investigating subgroups within the hRI group. Evidence suggests that there are impairments in EVT behaviour and spiral artery remodelling in the hRI group [21], [22]. Further definition of this group may allow us to study firstly those with a very high likelihood of developing PE and secondly those where the initial impairment in remodelling is not sufficient to lead to the clinical condition. In this second group we could postulate that this is because remodelling was merely delayed and there are compensatory mechanisms which overcome this (here the considerable overlapping roles that fetal trophoblast and the maternal immune cells have during remodelling may be highly relevant). Additionally there are likely to be inherent differences in how different mothers respond to factors produced by an intermittently perfused and stressed placenta, perhaps reflecting differences at a cardiovascular level [28]. This group is particularly interesting as it may help us to define how some women are more able to tolerate or compensate for early problems. It is only when we can grasp this that we can consider how we might therapeutically make one of the groups resemble the other in outcome. This is summarised in Fig. 2.

The complexities of modelling cellular interactions in a more 3-dimensional (3D) environment also provide challenges and potential for development. Immunohistochemical studies can provide much information about early human pregnancy however *in vitro* culture systems are needed to look at dynamic multi-cellular interactions. Some of the general processes involved in spiral artery remodelling have been reported by a number of groups, including our own, using monolayer co-cultures [29] and explant cultures [18], [30], and have revealed roles for EVT-dependent apoptotic vascular cell loss [19], [31], VSMC de-differentiation [32] and disruption of cellular interactions through proteases [33]. Growing vascular cells in a 3D spheroidal model that recapitulates EC/VSMC interactions seen *in vivo* allowed us to determine which genes were differentially expressed following stimulation by trophoblast and show VSMC de-differentiation [34]. The future application of some of the newer 3D technologies, many of which stem from developments in the cancer field, will allow even more accurate placental and decidual modelling. These developments include sophisticated synthetic matrices and scaffolds and approaches such as Real Architecture for 3D tissues (RAFT) technology [35].

Advances in isolation and culture methods for primary cells means that multiple cell types can be isolated from first trimester tissue from an individual pregnancy. We routinely isolate stromal cells, dNK cells and macrophages as well as endothelial cells (which will include those from spiral arteries) from decidual tissue of the uterine artery Doppler screened women. From the placenta we can isolate trophoblast, stromal, endothelial and macrophages. In the future the ability to look at multiple cell types across an individual pregnancy (with a known risk of developing complications) provides an opportunity for some complex profiling integrating information about genes, microRNAs, proteome and secretome with readouts from functional and biochemical cell based assays. This will allow us to start to get an overview of the maternal-fetal interface in an individual pregnancy. Determining whether this complex information can be integrated to model both normal and PE pregnancies will require strong collaborations with the bioinformatics and mathematical modelling fields. We will then be able to interrogate these models asking questions such as the importance of particular cellular or molecular interactions to successful placentation. The ultimate aim of developing robust models of the maternal-fetal interface would be to help in the identification of novel targets and the safe design of therapies.

## Conflict of interest

There is no conflict of interests.

## References

[1] Pijnenborg R., Vercruysse L., Hanssens M. (2006). The uterine spiral arteries in human pregnancy: facts and controversies. Placenta.

[2] Kam E.P., Gardner L., Loke Y.W., King A. (1999). The role of trophoblast in the physiological change in decidual spiral arteries. Hum. Reprod..

[3] Pijnenborg R., Vercruysse L., Brosens I. (2011). Deep placentation. Best. Pract. Res. Clin. Obstet. Gynaecol..

[4] Gairhe S., Bauer N.N., Gebb S.A., McMurtry I.F. (2011). Myoendothelial gap junctional signaling induces differentiation of pulmonary arterial smooth muscle cells. Am. J. Physiol. Lung Cell Mol. Physiol..

[5] Owens G.K. (2007). Molecular control of vascular smooth muscle cell differentiation and phenotypic plasticity. Novartis Found. Symp..

[6] Smith S.D., Dunk C.E., Aplin J.D., Harris L.K., Jones R.L. (2009). Evidence for immune cell involvement in decidual spiral arteriole remodeling in early human pregnancy. Am. J. Pathol..

[7] Fraser R., Whitley G.S., Johnstone A.P., Host A.J., Sebire N.J., Thilaganathan B., Cartwright J.E. (2012). Impaired decidual natural killer cell regulation of vascular remodelling in early human pregnancies with high uterine artery resistance. J. Pathol..

[8] Fraser R., Whitley G.S., Thilaganathan B., Cartwright J.E. (2015). Decidual natural killer cells regulate vessel stability: implications for impaired spiral artery remodelling. J. Reprod. Immunol..

[9] Lash G.E., Pitman H., Morgan H.L., Innes B.A., Agwu C.N., Bulmer J.N. (2016 Aug). Decidual macrophages: key regulators of vascular remodeling in human pregnancy. J. Leukoc. Biol..

[10] Robson A., Harris L.K., Innes B.A., Lash G.E., Aljunaidy M.M., Aplin J.D., Baker P.N., Robson S.C., Bulmer J.N. (2012). Uterine natural killer cells initiate spiral artery remodeling in human pregnancy. Faseb J..

[11] Hazan A.D., Smith S.D., Jones R.L., Whittle W., Lye S.J., Dunk C.E. (2010). Vascular-leukocyte interactions: mechanisms of human decidual spiral artery remodeling in vitro. Am. J. Pathol..

[12] Wallace A.E., Fraser R., Cartwright J.E. (2012). Extravillous trophoblast and decidual natural killer cells: a remodelling partnership. Hum. Reprod. Update.

[13] Carter A.M. (2007). Animal models of human placentation–a review. Placenta.

[14] Founds S.A., Conley Y.P., Lyons-Weiler J.F., Jeyabalan A., Hogge W.A., Conrad K.P. (2009). Altered global gene expression in first trimester placentas of women destined to develop preeclampsia. Placenta.

[15] Rabaglino M.B., Post Uiterweer E.D., Jeyabalan A., Hogge W.A., Conrad K.P. (2015). Bioinformatics approach reveals evidence for impaired endometrial maturation before and during early pregnancy in women who developed preeclampsia. Hypertension.

[16] Conrada Kirk P., Rabaglinob Maria Belen, Post Uiterweerc Emiel D. (2017). Emerging Role for Dysregulated Decidualization in the Genesis of Preeclampsia.

[17] Peters D.G., Yatsenko S.A., Surti U., Rajkovic A. (2015). Recent advances of genomic testing in perinatal medicine. Seminars perinatology.

[18] Cartwright J.E., Kenny L.C., Dash P.R., Crocker I.P., Aplin J.D., Baker P.N., Whitley G.S. (2002). Trophoblast invasion of spiral arteries: a novel in vitro model. Placenta.

[19] Ashton S.V., Whitley G.S., Dash P.R., Wareing M., Crocker I.P., Baker P.N., Cartwright J.E. (2005). Uterine spiral artery remodeling involves endothelial apoptosis induced by extravillous trophoblasts through Fas/FasL interactions. Arteriosclerosis, thrombosis, Vasc. Biol..

[20] Leslie K., Whitley G.S., Herse F., Dechend R., Ashton S.V., Laing K., Thilaganathan B., Cartwright J.E. (2015). Increased apoptosis, altered oxygen signaling, and antioxidant defenses in first-trimester pregnancies with high-resistance uterine artery blood flow. Am. J. Pathol..

[21] Whitley G.S., Dash P.R., Ayling L.J., Prefumo F., Thilaganathan B., Cartwright J.E. (2007). Increased apoptosis in first trimester extravillous trophoblasts from pregnancies at higher risk of developing preeclampsia. Am. J. Pathol..

[22] Prefumo F., Sebire N.J., Thilaganathan B. (2004). Decreased endovascular trophoblast invasion in first trimester pregnancies with high-resistance uterine artery Doppler indices. Hum. Reprod..

[23] Wallace A.E., Fraser R., Gurung S., Goulwara S.S., Whitley G.S., Johnstone A.P., Cartwright J.E. (2014). Increased angiogenic factor secretion by decidual natural killer cells from pregnancies with high uterine artery resistance alters trophoblast function. Hum. Reprod..

[24] Wallace A.E., Host A.J., Whitley G.S., Cartwright J.E. (2013). Decidual natural killer cell interactions with trophoblasts are impaired in pregnancies at increased risk of preeclampsia. Am. J. Pathol..

[25] Wallace A.E., Whitley G.S., Thilaganathan B., Cartwright J.E. (2015). Decidual natural killer cell receptor expression is altered in pregnancies with impaired vascular remodeling and a higher risk of pre-eclampsia. J. Leukoc. Biol..

[26] Smith S.D., Choudhury R.H., Matos P., Horn J.A., Lye S.J., Dunk C.E., Aplin J.D., Jones R.L., Harris L.K. (2015). Changes in vascular extracellular matrix composition during decidual spiral arteriole remodeling in early human pregnancy. Histology Histopathol..

[27] O'Gorman N., Wright D., Poon L.C., Rolnik D.L., Syngelaki A., Wright A., Akolekar R., Cicero S., Janga D., Jani J., Molina F.S., de Paco Matallana C., Papantoniou N., Persico N., Plasencia W., Singh M., Nicolaides K.H. (2017 Jan 9). Accuracy of competing risks model in screening for pre-eclampsia by maternal factors and biomarkers at 11-13 weeks' gestation. Ultrasound Obstet. Gynecol..

[28] Melchiorre K., Sharma R., Thilaganathan B. (2014). Cardiovascular implications in preeclampsia: an overview. Circulation.

[29] Chen Q., Stone P.R., McCowan L.M., Chamley L.W. (2005). Interaction of Jar choriocarcinoma cells with endothelial cell monolayers. Placenta.

[30] Dunk C., Petkovic L., Baczyk D., Rossant J., Winterhager E., Lye S.J. (2003). A novel in vitro model of trophoblast-mediated decidual blood vessel remodeling. Lab. Investig..

[31] Harris L.K., Keogh R.J., Wareing M., Baker P.N., Cartwright J.E., Aplin J.D., Whitley G.S. (2006). Invasive trophoblasts stimulate vascular smooth muscle cell apoptosis by a fas ligand-dependent mechanism. Am. J. Pathol..

[32] Bulmer J.N., Innes B.A., Levey J., Robson S.C., Lash G.E. (2012). The role of vascular smooth muscle cell apoptosis and migration during uterine spiral artery remodeling in normal human pregnancy. Faseb J..

[33] Harris L.K., Smith S.D., Keogh R.J., Jones R.L., Baker P.N., Knofler M., Cartwright J.E., Whitley G.S., Aplin J.D. (2010). Trophoblast- and vascular smooth muscle cell-derived MMP-12 mediates elastolysis during uterine spiral artery remodeling. Am. J. Pathol..

[34] Wallace A.E., Cartwright J.E., Begum R., Laing K., Thilaganathan B., Whitley G.S. (2013). Trophoblast-induced changes in C-x-C motif chemokine 10 expression contribute to vascular smooth muscle cell dedifferentiation during spiral artery remodeling. Arteriosclerosis, thrombosis, Vasc. Biol..

[35] Levis H.J., Kureshi A.K., Massie I., Morgan L., Vernon A.J., Daniels J.T. (2015). Tissue engineering the cornea: the evolution of RAFT. J. Funct. biomaterials.

